# Adsorption of adenine on mercury electrode in acetate buffer at pH 5 and pH 6 and its effect on electroreduction of zinc ions

**DOI:** 10.1007/s00706-018-2183-1

**Published:** 2018-06-28

**Authors:** Dorota Gugała-Fekner

**Affiliations:** Faculty of Chemistry, Maria Curie-Sklodowska University, Lublin, Poland

**Keywords:** Electrochemistry, Differential capacity, Surface, Adsorption isotherm, Electrosorption

## Abstract

**Abstract:**

The measurements of double-layer differential capacitance, zero charge potential, and surface tension at that potential allowed us to examine the adsorption properties of adenine on the mercury surface from the neat buffer solution, i.e., the acetate buffer at pH 5 and pH 6. The systems obtained at such pH values were close to physiological fluids in their characteristics. The adsorption energy and interaction constants were determined using Frumkin isotherm and virial isotherm. It was shown that the adenine molecule is adsorbed on the mercury electrode with its negative pole against the electrode surface. Using the cyclic voltammetry technique and measuring Faraday impedance, an increasing effect of adenine on the kinetics of zinc ion electroreduction was found. In both buffer solutions, the neutral adenine molecules can form on the surface of the working electrode, an unstable active complex with depolarizer ions, facilitating electron exchange.

**Graphical abstract:**

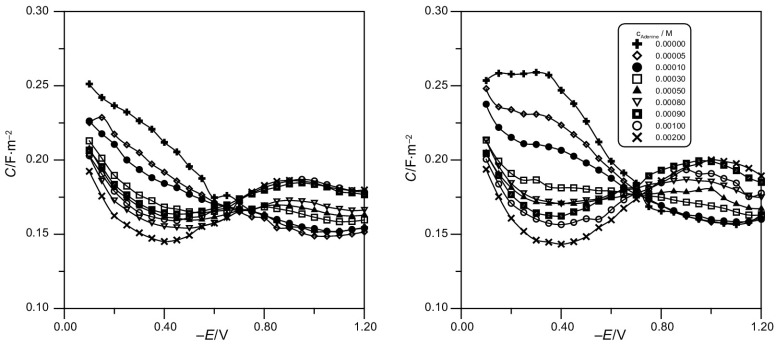

## Introduction

In recent years, a rapid development of genetics has been observed, which is an inspiration for thorough studies on the basic units that create the structure of nucleic acids [1, 2]. Adenine is a purine derivative; 5-aminopurine is one of five nitrogenous bases that are used in construction of nucleotides, which in turn build up the nucleic acids such as DNA, RNA, and some coenzymes, e.g., NAD. The charged surfaces near the boundary between biopolymers and cell fluids are regarded as the systems that are comparable to the adsorbate on the electrode in the field of the electrical double layer [3].

Adenine, which was selected for the present investigations, occurs in the free state in tea leaves, yeast, and fungi. The aromatic character of adenine points to a possible mechanism of adsorption on the electrode surface [4]. Particularly, at its low concentrations in solution, one can expect a flat orientation of the adenine molecule on the electrode surface. On the other hand, the presence of the –NH_2_ group indicates that adenine exhibits the properties of complex formation.

The mercury electrode is most frequently used in investigations of organic compounds because of a very large polarizability range and the benefit of providing repeatable and accurate results [5, 6]. A very good reproducibility of the results obtained on the dropping mercury electrode is due to the homogeneity and purity of the mercury-solution boundary surface as compared with solid electrodes as well as the possibility of using different measurement techniques, in particular the measurements of differential capacitance of the double layer and surface tension, which allow one to obtain accurate experimental data [7–9].

The acetate buffers pH 5 and pH 6 were used, because the acetate ions are weakly adsorbed on the mercury electrode. This allows omitting the competitive adsorption of these ions with molecules or ions of organic substances affecting the kinetics of the electrode reaction.

Measurements were made at low pH due to the avoidance of Zn^2+^ ion hydrolysis.

## Results and discussion

The adsorption at the metal–solution interface is described by the following parameters: the energy of adsorption, the interaction constant, and surface excess which reflects the surface concentration of the adsorbate. The values of the above parameters can be calculated using the experimental data obtained by several methods. Among the methods that are most frequently applied and afford the best information about the structure of the electrical double layer and the adsorption at the metal–electrolyte solution interface are the measurements of the double-layer differential capacitance, zero charge potential, and surface tension. The data obtained from the measurements of differential capacitance allow one to qualitatively determine the state of the electrode surface. From the course of differential capacitance curves, one can deduce the presence of adsorbed molecules or ions [5, 10–13]. To measure the differential capacitance, we used the dropping mercury electrode with constant drop surface which was obtained by mechanical isolation of the drop after 6 s. To establish if the adsorption equilibrium was obtained in the range of the applied concentrations of organic substances, we performed the measurements of differential capacitance against the alternating current frequency.

**Fig. 1:**
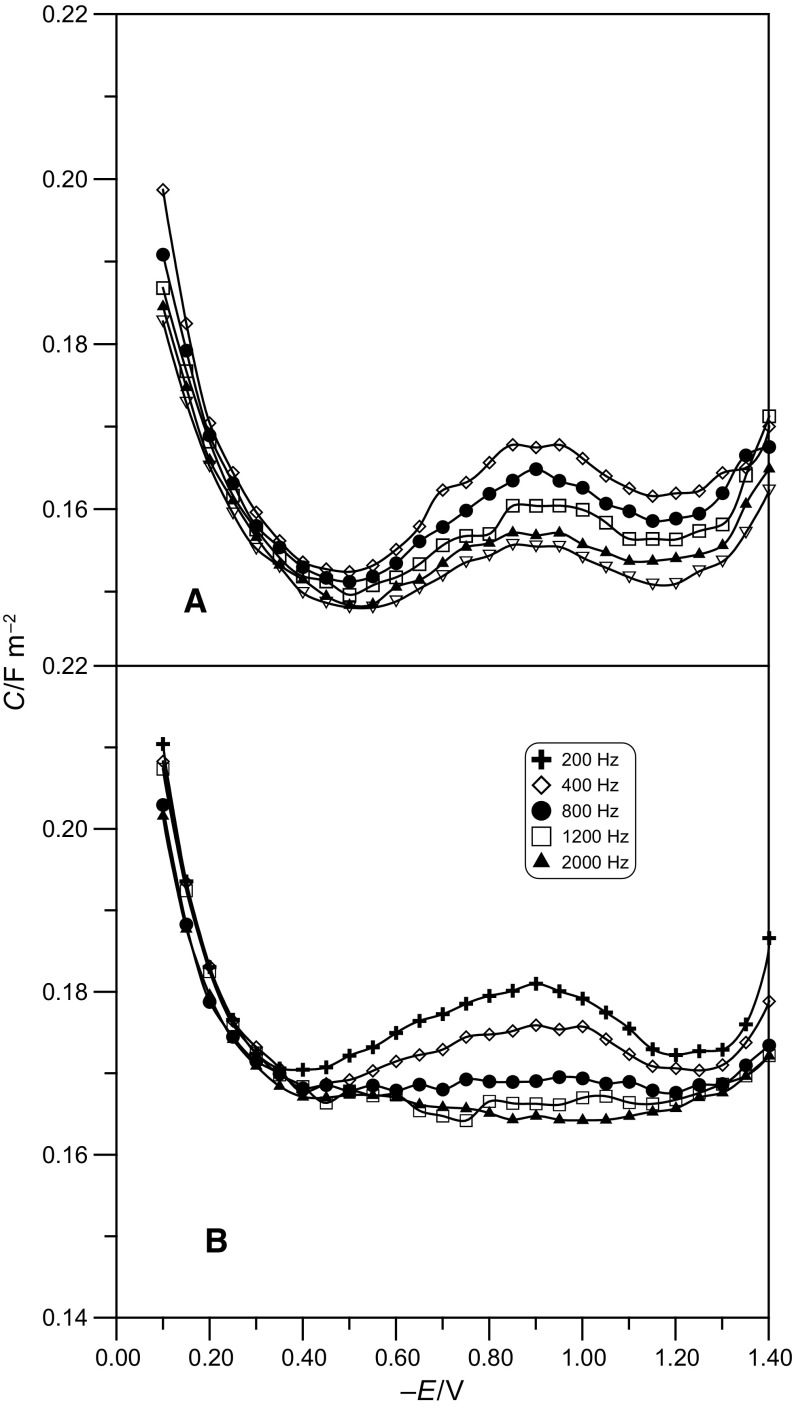
Dependence of differential capacitance on frequency for the solution of acetate buffer at pH 5 (**a**) and pH 6 (**b**) + 8 × 10^−3^ M adenine

Figure 1 presents examples of the dependence of double-layer differential capacitance curves on the electrode potential and the alternating current frequency for the acetate buffer + 8 × 10^−3^ M adenine system. The analysis of Fig. 1 shows that frequency dispersion occurs over the whole potential area, that is why the differential capacitance curves were obtained on the basis of the relationship *C *=* f* (*ω*) as a result of extrapolating the differential capacitances measured for the subsequent alternating current frequency values to the angular frequency: *ω* = 0. Then, the impedance of the double layer is presented as a model of resistance and capacity arranged in series, whereas the adsorption rate is diffusion controlled. A similar dependence was shown for all applied adenine concentrations.

**Fig. 2:**
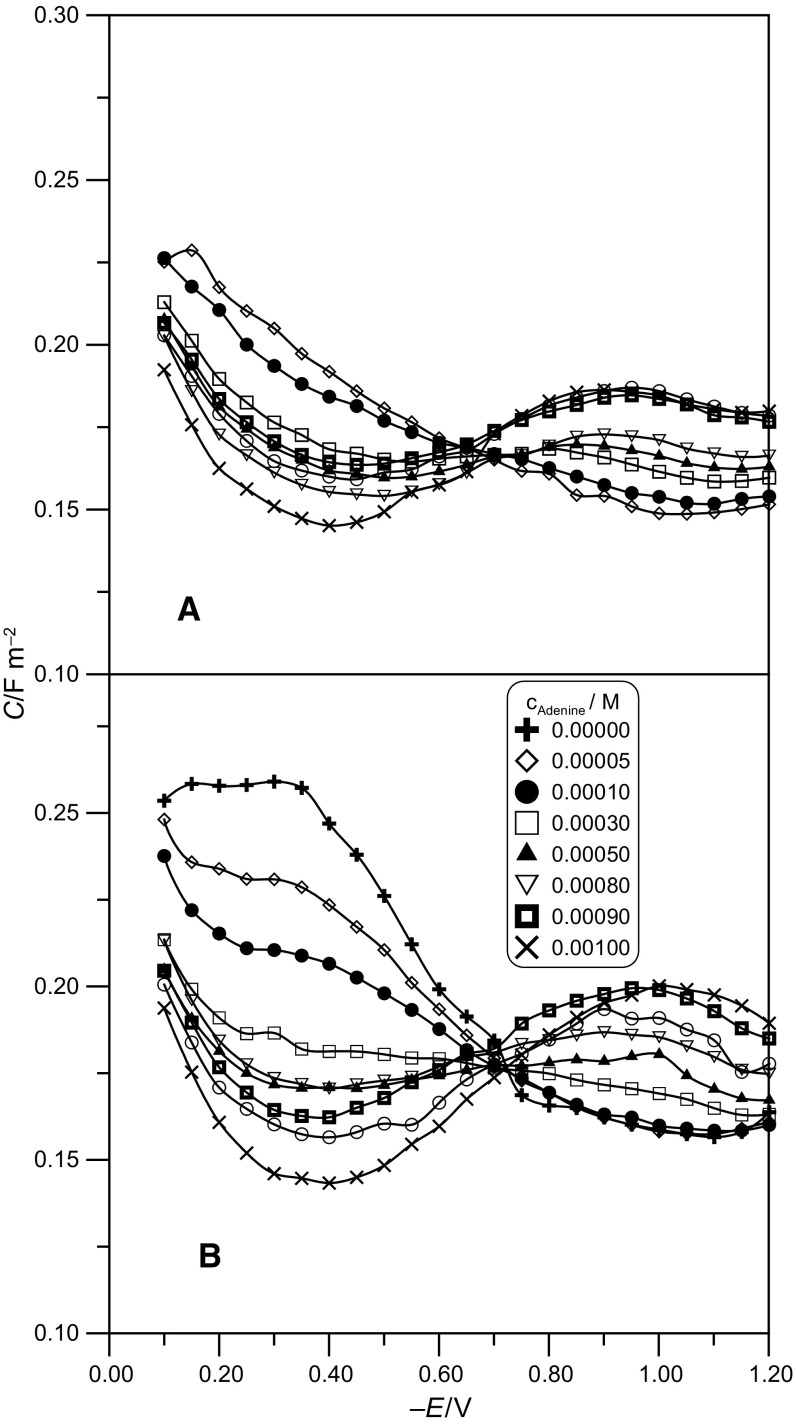
Differential capacitance-potential curves at Hg/acetate buffer at pH 5 (**a**) and pH 6 (**b**) for various adenine concentrations as shown in the figure legend

Figure 2 presents the curves of differential capacitance extrapolated to zero frequency in solutions at pH 5 and pH 6 containing different amounts of adenine in the potential range from *E * = − 0.10 V to *E * = − 1.20 V.

If we compare the course of differential capacitance curves for the solutions at pH 5 and pH 6 containing increasing adenine concentrations, we can distinguish two areas of potential: − 0.10 V <* E * < − 0.70 V and − 0.70 V <* E * < − 1.20 V.

In the first area, there is a gradual reduction of differential capacitance together with the increase of concentration of the organic substance. This reduction is a little stronger in the acetate buffer at pH 6. At the potential of about *E * = − 0.4 V, the maximum concentration of adenine has the strongest reducing effect on differential capacitance. Further analysis shows that, at the potential of about *E * = − 0.70 V, the curves of differential capacitance for both buffers intersect each other.

In the second area, the increase of concentration of the organic substance results in a considerable increase of differential capacitance value as compared with the values obtained for the neat buffer solution. In this area of potentials, a characteristic “hump” appears for the highest adenine concentrations, the height of which increases together with the increase of concentration of the substance. This “hump” coincides with a rise of differential capacitance in the area of more negative potentials.

The occurrence of this capacitance “hump” which is characteristic of strongly adsorbing anions or polar substances can be due to the changes in mutual interactions, mainly electrostatic ones, between the adsorbed molecules [14]. These changes can also cause a change of orientation in the adsorbed organic molecules. Undoubtedly, at the potentials where the capacitance “hump” appears, the adsorbate molecules are more weakly connected with the electrode surface than at the potentials at which there is reduction of differential capacitance with respect to differential capacitance of the neat buffer solution.

Thermodynamic description of adsorption requires performing integration of differential capacitance curves. If, for the whole range of concentrations, these curves coincide with the curve for neat buffer solution at negative enough potentials, then the parameters established for this electrolyte at fixed negative potential can be the constants of integration. In the examined system, such a phenomenon did not occur, so the values of zero charge potential, *E*_*z*_, and the surface tension, *γ*_*z*_ had to be determined as the constants of integration. At this potential, the electrical double layer practically does not exist if the ions are not specifically adsorbed.

Table 1 presents the values of zero charge potential, *E*_*z*_, and the surface tension, *γ*_*z*_, measured at *E*_*z*_ for the investigated systems. The *E*_*z*_ values in buffer solutions of increasing pH and not containing adenine are shifted towards negative potentials, which are related to a higher concentration of the acetate anions. The introduction of adenine to both neat buffers solutions results in shifting the *E*_*z*_ values mostly towards more negative potentials. These changes indicate that the organic molecule is adsorbed on the electrode with its negative pole, i.e., with the aromatic ring. The surface tensions measured at zero charge potential decrease with an increase of adenine concentration.

**Table 1:** Values of zero charge potentials *E*_*z*_ vs. Ag/AgCl electrode and surface tension *γ*_*z*_ (mN m^−1^) at *E*_*z*_ for the studied systems

*c*/10^−5^ M	pH 5		pH 6	
	− *E*_*z*_*/*V	*γ*_*z*_/mN m^−1^	− *E*_*z*_*/*V	*γ*_*z*_/mN m^−1^
0	0.416	419	0.422	431
5.0	0.387	472	0.425	470
10.0	0.397	462	0.425	462
30.0	0.397	462	0.431	460
50.0	0.398	462	0.438	458
80.0	0.417	457	0.448	456
90.0	0.419	457	0.454	455
100.0	0.419	451	0.460	453
200.0	0.414	448	0.474	448

The results of measurements of zero charge potential and the surface tension with that potential served as the constant of integration for differential capacitance curves. On the basis of the dependence (1), the charge density on the electrode surface was obtained:

1 $$\sigma = \mathop \int \limits_{{E_{z} }}^{E} C{\text{d}}E.$$

Twofold integration of differential capacitance curves was used to calculate the value of surface tension according to Eq. (2):

2 $$\gamma = \gamma_{z} - \mathop \int \limits_{{E_{z} }}^{E} \mathop \smallint \nolimits C{\text{d}}E.$$

The obtained values of surface charge were used to determine the parameters characterizing maximum adsorption: the potential of maximum adsorption *E*_max_ and the charge of maximum adsorption *σ*_max_ (Table 2). The possibility of accurate determination of *E*_max_ and *σ*_max_ indicates a physical nature of adenine adsorption (physisorption).

**Table 2:** Values of the charge and potential at which the maximum of adsorption determined for the solutions at pH 5 and pH 6 is obtained

	pH 5	pH 6
$\sigma /10^{ - 2} {\text{C m}}^{ - 2}$	1.18	1.61
− *E*_max_/V	0.35	0.35

### Adsorption isotherms

The adsorption of adenine was described using relative surface excess, *Г*′, which, according to the Gibbs adsorption isotherm [15], is given by the following:

3 $$\varGamma^{\prime} = \frac{1}{RT}\left( {\frac{\partial \varPhi }{{\partial { \ln }c}}} \right)_{E} ,$$

where *c* is the bulk concentration of adenine and *Φ* is surface tension, as in *Φ* = *γ*_0 _− *γ*, where *γ*_0_ is the surface tension of the neat buffer solution, and *γ* is the surface tension for the solution containing adenine.

**Fig. 3:**
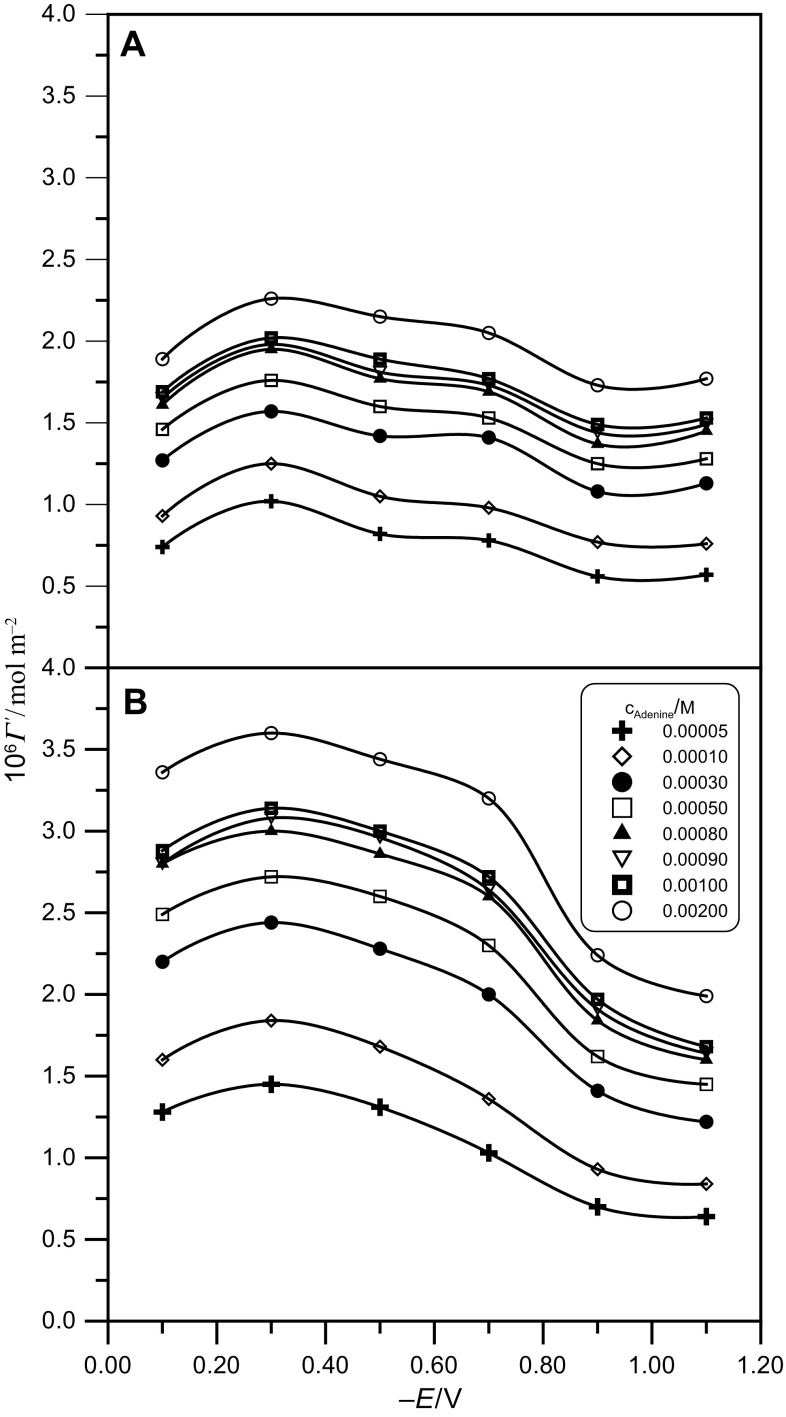
Relative surface excess of adenine as a function of potential and adenine concentration in the bulk, in the acetic buffers at pH 5 (**a**) and pH 6 (**b**)

The values of adenine surface concentrations expressed as relative surface excess were analyzed to obtain a more complete picture of adsorption of the tested substance (Fig. 3).

The shape of the curves in Fig. 3 seems to indicate that there are competitive electrostatic interactions between the adenine molecules, acetate ions, acetic acid molecules, and water dipoles [16]. Along with the increase of adenine concentration in the examined systems, an increase of surface excess was observed at the given electrode potential. The obtained values of *Γ*^′^ for adenine are a little higher in the buffer at pH 6. The maximum *Γ*^′^ values were observed near the potential of maximum adsorption. The shape of the *Γ*^′^ = *f*(E) curves with a marked maximum resembles respective dependences for a number of aliphatic compounds, which seems to indicate a physical character of the adsorption of adenine on the mercury electrode.

Using the obtained *Γ*^′^ values for the determined electrode potentials and adenine concentrations, the linear Frumkin isothermal test was determined based on the equation:

4 $$\beta x = \left[ {\theta /\left( {1 - \theta } \right)} \right]\exp \left( { - 2A\theta } \right),$$

where *x* is the mole fraction of adenine in solution, *β* is the adsorption coefficient which is defined as *β* = exp(−*ΔG*_F_^o^/*RT*), and *θ* is the surface coverage *θ* = *Γ*^′^/*Γ*_s_, $\Delta G_{\text{F}}^{\text{o}}$ is the free energy of adsorption.

The surface excess at saturation, *Γ*_s_, was estimated by extrapolation of the 1/*Γ*^′^ vs. 1/*c*_adenine_ lines at different electrode potentials to $1/c_{\text{adenine}} = 0$. The surface occupied by one adenine molecule, *S* (*S* = 1/*Γ*_*s*_) was 0.49 nm^2^ for pH 5 and 0.33 nm^2^ for pH 6. The *Γ*_s_ value obtained in this way was 3.33 × 10^−6^ mol m^−2^ for the buffer at pH 5 and pH 6, respectively. The increase of pH of the neat buffer solution results in an increase of *Γ*_s_ value. This can indicate that the adsorption of adenine is facilitated due to a higher concentration of acetate ions.

**Fig. 4:**
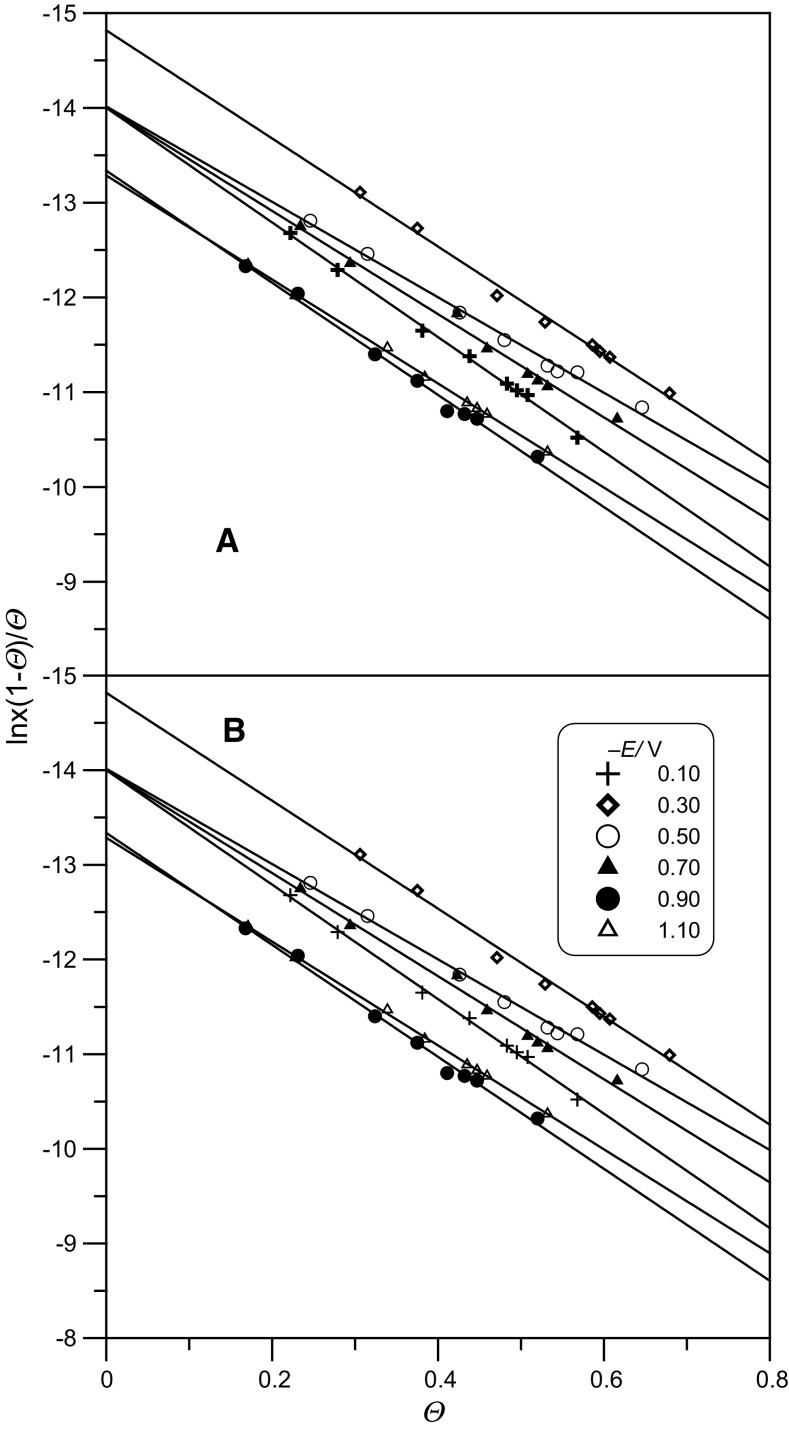
Linear test of the Frumkin isotherm in the system: the acetic buffer at pH 5 (**a**) and pH 6 (**b**) + adenine for different electrode potentials

Figure 4 presents the Frumkin isotherm test for the studied solutions. The increase of pH of the neat buffer solution is accompanied by an increase of the degree of coating, *θ*. Table 3 presents the values of the free energy of adsorption, Δ*G*_F_^o^, and the interaction constant, *A*, obtained from linear isotherm tests against the electrode potential. The values of parameter *A* were calculated from the slopes of particular lines shown in Fig. 4, whereas the values of the free energy of adsorption Δ*G*_F_^o^ were established by their extrapolation to the value *θ* = 0.

**Table 3:** Constants of Frumkin (F) and virial (V) isotherms for the system: the acetic buffer at pH 5 and pH 6 + adenine

− *E*/mV	pH 5				pH 6			
	Δ*G*_F_^o^/kJ mol^−1^	− *A*_F_	Δ*G*_V_^o^/kJ mol^−1^	*B*/nm^2^	Δ*G*_F_^o^ /kJ mol^−1^	− *A*_F_	Δ*G*_V_^o^/kJ mol^−1^	*B*/nm^2^
100	34.68	3.03	112.4	1.66	34.78	2.44	113.6	0.95
300	36.73	2.83	115.1	1.77	35.07	2.30	119.2	0.96
500	34.68	2.47	112.4	1.46	35.67	2.32	113.7	0.91
700	34.68	2.72	112.4	1.56	33.24	2.14	112.0	0.86
900	33.09	3.08	110.6	1.59	32.20	3.22	110.7	1.12
1100	32.75	2.69	110.6	1.52	32.20	4.00	110.7	1.37

From the presented adsorption parameters, it results that the energy of adsorption Δ*G*_F_^o^ for adenine is comparable in both buffer solutions and is accompanied by repulsive interactions between the adsorbed adenine molecules. The Δ*G*_F_^o^ values increase together with the increase of electrode potential, which is confirmed by the mechanism of the adsorption of adenine with its negative pole directed to the electrode surface. The values of parameter *A* indicate that the effect of the electrode potential on repulsive interaction between the adsorbed adenine molecules in the buffer at pH 5 is very small. In *E* > 0.70 V, the increase of repulsive interactions in this buffer for the lowest electrode potentials seems to explain a sudden decrease of *Γ*^′^ at these potentials.

**Fig. 5:**
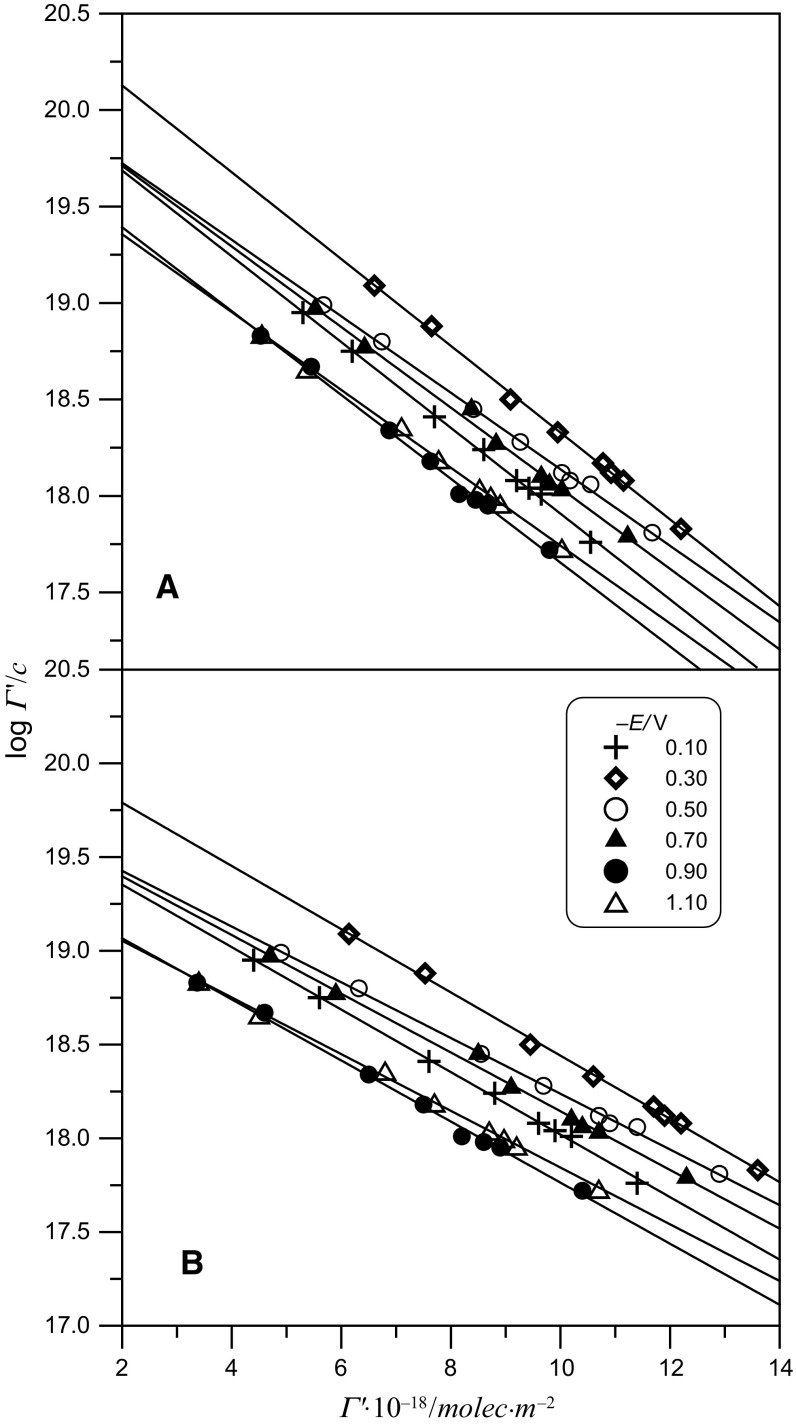
Linear test of the virial isotherm in the system: acetic buffer at pH 5 (**a**) and pH 6 (**b**) + adenine for different electrode potentials

The previously presented Frumkin isotherms are encumbered with errors resulting from discrepancies between theoretical and experimental values of *Γ*_s_. To confirm the obtained results, a test was performed using the virial isotherm in which parameter *Γ*_s_ does not exist. The virial isotherm equation is as follows:

5 $$\ln \beta c = \ln \varGamma + 2B\varGamma ,$$

where *β* is a two-dimensional (2D) second virial coefficient. The linear test for the virial isotherm was performed in the system: log $\left( {\varGamma^{\prime}/c} \right){\text{vs}}. \;\varGamma^{\prime}$ using the standard state 1 mol dm^−3^ in the bulk solution and 1 mol cm^−2^ on the surface (Fig. 5).

The presented adsorption parameters obtained from the virial isotherm (Fig. 5, Table 3) confirmed the values of adsorption parameters obtained from the Frumkin isotherm. On the basis of these parameters, it can be said that weaker repulsive interactions between the adenine molecules are responsible for higher *Γ*^′^ values obtained for adenine in the buffer at pH 6. This is clearly shown by smaller values of parameter *B* obtained in this solution. Such a dependence was not observed on the basis of parameter − *A*_F_. These weaker repulsive interactions can result from the shielding of positive dipole poles of the adenine molecules by acetate ions, whose concentration in the buffer at pH 6 is a little higher than in the buffer at pH 5.

### The effect of adenine on the kinetics of reduction of zinc ions in acetate buffer at pH 5 and pH 6

The investigations on the effect of adenine on the kinetics of reduction of zinc ions were performed using cyclic voltammetry (CV) and Faraday impedance techniques.

Using the method of single-sweep voltammetry, the potential between the working electrode and the counter electrode is swept linearly in time. Its characteristic feature is that after exceeding the peak potential, the electrode potential proceeds in the reverse direction [17–20]. During the studies performed by cyclic voltammetry, the electrode was polarized by a linear potential changing in time in the range from *E * = − 0.80 V to *E * = − 1.10 V and the current intensity proceeding in the function of electrode potential was registered. In this way, the voltammetric curves were obtained on which two peaks could be distinguished. One of them is the cathodic peak that appears at more negative potential values. The reversed direction of potential changes results in anodic oxidation of the product precipitated on the electrode. Then, the other peak is formed, which is the anodic peak.

On the basis of CV voltampergrams, the values of cathodic and anodic peak potentials were determined (*E*_a_ i *E*_c_). Their knowledge allowed me to calculate the value the reversible half-wave potential $E_{ 1 / 2}^{\text{r}}$ of Zn^2+^ in the absence and presence of adenine [21]. As can be seen from Table 4, the increase in adenine concentration does not cause significant changes in $E_{ 1 / 2}^{\text{r}}$ values in acetate buffer at pH 5 and pH 6. It can, therefore, be concluded that the complexes between the ions of the depolarizer and the organic substance are not permanently formed in the solution.

**Table 4:** Values of potentials for the anodic and cathodic peaks, differences between them, and the reversible half-wave potential of Zn^2+^ reduction for the system: 5 × 10^−3^ M zinc ions in buffer solutions at pH 5 and pH 6 without adenine and for various adenine concentrations

*c*/10^−5^ M	pH 5				pH 6			
	− *E*_a_*/*mV	− *E*_c_*/*mV	Δ*E/*mV	$E_{ 1 / 2}^{\text{r}} /{\text{mV}}$	− *E*_a_*/*mV	− *E*_c_*/*mV	Δ*E/*mV	$E_{ 1 / 2}^{\text{r}} /{\text{mV}}$
0	− 951	− 1010	59	− 980	− 954	− 1018	64	− 986
5.0	− 952	− 1011	60	− 981	− 958	− 1020	62	− 984
10.0	− 951	− 1012	62	− 982	− 954	− 1014	60	− 984
30.0	− 952	− 1010	58	− 981	− 959	− 1014	55	− 986
50.0	− 952	− 1008	55	− 980	− 959	− 1010	51	− 984
80.0	− 954	− 1008	54	− 981	− 959	− 1010	51	− 984
90.0	− 954	− 1008	54	− 981	− 960	− 1010	50	− 985
100.0	− 954	− 1008	54	− 981	− 960	− 1009	49	− 984
200.0	− 954	− 1008	54	− 981	− 962	− 1010	48	− 986
300.0	− 954	− 1008	54	− 981	− 960	− 1007	47	− 983
400.0	− 954	− 1008	54	− 981	− 962	− 1006	44	− 984
500.0	− 955	− 1008	53	− 981	− 962	− 1004	42	− 983

On the basis of the changes of position of the cathodic and anodic peaks due to the introduction of adenine to the examined solutions, the values of potential difference between the anodic peak and the cathodic peak, Δ*E*, were calculated based on the dependence (6):

6 $$\Delta E = E_{\text{a}} - E_{\text{c}} .$$

The potential differences between the anodic and cathodic peaks are a good criterion to evaluate the reversibility of the electrode process. The Δ*E* values determined for the examined systems are presented in Table 4.

On the basis of data presented in Table 4, it can be said that the addition of adenine has an accelerating effect on the process of zinc reduction on the mercury electrode. This catalytic ability of adenine is definitely greater at a higher pH value.

In the described method, the working electrode was polarized with a constant current while modulating the voltage in the range of frequencies 25–100,000 Hz at potentials ranging from *E * = − 0.91 V to *E * = − 1.06 V. The polarization potential was changed every 10 mV. For each applied polarization potential, the activation resistance, *R*_*a*_, was determined. Comparing the values of minimum activation resistance determined for particular experimental systems, we could observe a decrease of the *R*_*a*_ value together with an increase of adenine concentration (Table 5). This demonstrates that the catalysis of zinc ions reduction occurs. The decrease is higher in solution at pH 6, which confirms a greater catalytic ability of adenine at this pH value. The accelerating effect of adenine on electroreduction of zinc ions in both acetate buffers may result from the fact that neutral adenine molecules with a lone electron pair at the nitrogen atom of the –NH_2_ group can form an unstable active complex with depolarizer ions on the electrode surface. Probably creation, this complex facilitates the exchange of electrons during the electrode process [22–25].

**Table 5:** Minimum values of activation resistance at the given potentials in the system: 5 × 10^−3^ M zinc ions in buffer solutions at pH 5 and pH 6 in the presence of increasing adenine concentrations

*c*/10^−5^ M	pH 5		pH 6	
	− *E/*mV	*R*_A_*/*Ω cm^2^	− *E/*mV	*R*_A_*/*Ω cm^2^
0	980	4.76	990	4.82
5.0	980	3.99	990	4.76
10.0	980	3.53	990	4.24
30.0	990	2.78	990	2.95
50.0	980	2.44	990	2.11
80.0	990	2.16	990	1.93
90.0	990	2.16	990	1.70
100.0	990	2.19	990	1.60
200.0	990	1.97	990	1.32
300.0	990	1.97	1000	1.17
400.0	990	2.02	1000	1.18
500.0	990	1.95	1000	1.12

## Conclusion

Based on experimental data as well as on the results of thermodynamic analysis concerning the adsorption of adenine on the mercury electrode, the following conclusions can be drawn.

The differential capacitance curves for adenine do not coincide with those obtained for the neat buffer solution. This is the evidence of adsorption of a given compound on the mercury electrode over the whole range of applied potentials. In the acetate buffer at pH 6 for *E* > − 0.6 V, the decrease of differential capacitance is greater than in the buffer at pH 5. This shows that the adsorption of adenine is stronger in the buffer at pH 6.

Together, with the increase of concentration of the organic substance, a shift of zero charge potential occurs towards more negative potentials, which indicates that the adenine molecule adsorbs on the electrode surface with its negative pole against the metal (aromatic ring). The surface tension measured at zero charge potential decreases together with an increase of adenine concentration.

The obtained values of this surface tension show that the adsorption of adenine is stronger at pH 6. The adsorption in this buffer is facilitated because of weaker hydration on the electrode surface that results from a greater concentration of the acetate ions.

In acetate buffers with pH 4 [26], 5, and 6, as the concentration of organic matter increases, the potential of the zero charge is shifted towards the more potential negative ones. This indicates that the adenine molecule adsorbs to the surface of the electrode with a negative pole or aromatic ring. In the pH 3 buffer, the adenine molecule is more strongly protonated and adsorbed to the positive mercury electrode [26].

The physical character of the adsorption of adenine on the mercury electrode is evidenced by: (1) the possibility of determination of the charge and maximum adsorption potential in both examined buffers, and (2) the fact that the curves showing the dependence of relative excess values against the potential are bell-shaped. A similar character of adsorption of adenine on the mercury electrode was observed in buffer with pH 4 [26] and guanine at pH 4 and 6 [27].

The investigation into the kinetics of electroreduction of zinc ions by cyclic voltammetry and Faraday impedance allowed us to conclude that, in both buffers, the addition of adenine accelerates the process of zinc reduction on the mercury electrode. The organic substance which was added to the solution acts as a catalyst and its activity is stronger at a higher pH of the applied buffers. In the case of Zn^2+^ ion electroreduction kinetics in pH 3 and 4 acetate buffers, it was noted that the reduction reactions in these buffers are irreversible. In pH 3 buffer, electroreduction is more irreversible than in pH 4 buffer [25].

## Experimental

Analytical grade adenine, acetic acid, and sodium acetate (Fluka) were used without further purification. Water and mercury were double distilled before use. The adenine solutions of concentrations, ranging from 5 × 10^−5^ to 2 × 10^−3^ M, were prepared immediately before every measurement. The chosen surfactant concentrations were lower than the surfactant critical micelle concentration. The solutions were deaerated by passing high purity nitrogen over the solution during the measurements, at 298 ± 0.1 K. A three-electrode system was used, with a dropping mercury electrode as a working electrode, an Ag/AgCl electrode as a reference electrode to which all potentials in this paper are referred, and a platinum electrode as an auxiliary electrode. A controlled growth mercury drop electrode (CGMDE), manufactured by MTM (Poland), was used. The differential capacitance, *C*, of the double layer was measured with an Autolab frequency response analyser, (Eco Chemie, The Netherlands), using the AC impedance technique. The measurements were carried out at several frequencies in the range from 400 to 2000 Hz, with an amplitude of 5 mV. The equilibrium capacities were obtained by extrapolation of the dependence of the measured capacity versus the square root of the frequency to zero frequency.

The potential of zero charge, *E*_*z*_, was measured using a streaming electrode. The interfacial tension, $\gamma_{z}$, at *E*_*z*_ was measured by the maximum bubble pressure method according to Schiffrin [28]. The charge density and surface tension were obtained by the back integration of the differential capacitance—potential dependences. No corrections were made for the effect of the medium on the activity of the supporting electrolyte [29, 30] and the activity coefficient of the adsorbate [31].

The zinc ion electroreduction studies were performed by the cyclic voltammetry and Faraday impedance measurement using an Autolab frequency response analyser. The experiments were performed in the system described earlier [20, 27].
